# Individual traits that influence the frequency and emotional characteristics of involuntary musical imagery: An experience sampling study

**DOI:** 10.1371/journal.pone.0234111

**Published:** 2020-06-04

**Authors:** Kazumasa Negishi, Takahiro Sekiguchi

**Affiliations:** Department of Educational Psychology, Tokyo Gakugei University, Koganei, Tokyo, Japan; Aalborg University, DENMARK

## Abstract

In this study, we investigated individual traits that influence the frequency of involuntary musical imagery (INMI) and the emotional valence of these occurrences using the experience sampling method (ESM) that measures INMI in daily life at the moment they occur. As individual traits, the effects of non-clinical obsessive-compulsive (OC) tendencies, personality traits, and musical expertise were examined. Among them, we were particularly interested in the effect of OC tendencies that are assumed to be related to INMI but are yet to be fully examined using ESM. A total of 101 university students completed questionnaires that assessed OC tendencies, the Big Five personality traits, and musical expertise. During the seven-day sampling period, participants received smartphone notifications six times per day and responded by stating whether they had experienced INMI and described the emotional characteristics of those occurrences. A multilevel analysis showed the relationship between OC tendencies and INMI. A positive effect was observed for intrusive thoughts (obsession) on the occurrence of INMI. Regarding the emotional characteristics of INMI, a negative effect of compulsive washing was observed on both the pleasantness of INMI experiences and the extent to which the participants liked the music they had heard internally. The effects of both personality traits and musical expertise were also observed in the analysis of INMI occurrences, both of which are consistent with previous findings. In summary, the present study using ESM supports previous findings on individual traits that affect INMI and clarifies them with additional detail and accuracy.

## Introduction

Involuntary musical imagery (INMI, colloquially called “earworm”) is a phenomenon in which an excerpt of music is perceived spontaneously in the absence of a corresponding external stimulus and repeats itself without conscious control. This phenomenon differs from pathological musical hallucinations in which music is heard vividly and experienced as if it were externally located [1], and is commonly observed in healthy populations. In a study based on an online survey, Liikkanen [2] reported that 89.2% of participants experienced INMI at least once a week, and 33.2% of them experienced it every day. Because of its ubiquitous and frequent nature, INMI is suitable for investigating basic features of involuntary aspects of human cognition as typified by involuntary autobiographical memory [3], mind wandering [4], and rumination [5].

INMI is often described as an unpleasant or even disturbing experience because of its uncontrollable repetitive presence in a person’s head [e.g., 6]. However, empirical research has suggested that the majority of INMI events are either neutral or pleasant experiences, and unpleasant INMI events occur only occasionally in daily life [7–10]. Meanwhile, there are individual differences in the feelings that accompany INMI [2, 7, 8, 11]. For example, Farrugia et al. [12] reported that the negative valence score of the Involuntary Musical Imagery Scale (IMIS) [11], which measures general tendency to feel INMI as disturbing and unpleasant experience, was widely distributed among individuals. This result indicates that while most people rarely experience INMI as negative, some people sometimes or often refer to it as a disturbing and unpleasant experience. Furthermore, there are also individual differences in how frequently INMI occurs in daily life [e.g., 2]. It is important to investigate the factors that influence emotional valence and the frequency of INMI to understand the nature and mechanism of this phenomenon.

In the present study, we examined the effects of individual traits on emotional characteristics and the frequency of INMI. Specifically, we focused on obsessive-compulsive (OC) tendencies, the Big Five personality traits, and musical expertise. Among them, we have a particular interest in the effects of OC tendencies. Obsession and compulsion are defining symptoms of obsessive-compulsive disorder (OCD). Obsessions are recurrent and persistent thoughts, urges, or impulses that are intrusively experienced with marked levels of anxiety or distress. Compulsions are repetitive behaviors (e.g., hand washing, ordering, checking) or mental acts (e.g., praying, counting, repeating words silently) that the individual feels driven to perform them in response to an obsession or according to rules that must be applied [13]. Both obsession and compulsion are not unique to OCD patients but are also present in the non-clinical population to varying degrees [14–16]. In this paper, we refer to this severity of the OC attributes in non-clinical people as OC tendencies, and examine the effect of OC tendencies on INMI experiences.

INMI is not regarded as a symptom of OCD because patients with OCD typically think of negative thoughts (e.g., contamination) and impulses rather than sensory input such as music. Meanwhile, some semi-academic books suggest that INMI is related to OCD because INMI is characterized by experiences where a perception of music playing repeatedly and persistently occurs in the head, similar to obsession [6, 17]. Therefore, it is natural to assume that OC tendencies are also associated with the frequency and unpleasantness of INMI. Indeed, there is reason to expect a positive relationship between the likelihood of INMI occurrence and OC tendencies. Previous studies have shown that INMI is more likely to occur during activities with a low cognitive load and that require low executive attention [9, 18, 19]. This suggests that INMI occurs when executive control works weakly. The studies of OCD have shown that the symptoms of OCD, and in particular obsession, are partially caused by poor executive control [20, 21]. Therefore, both INMI and the symptoms of OCD are suggested to be the result of faulty executive control. The relationship between OC tendencies and the unpleasantness of the INMI experience is also expected for the following reason. Individuals with high OC tendencies suffer from repetitive thoughts that occur unintentionally. From this perspective, it is plausible that they are likely to feel negative about the characteristics of INMI that occur repeatedly regardless of their intention, even though listening to music in their head itself is a pleasant experience.

To date, only two studies have examined whether OC tendencies really affect the characteristics of the INMI experience. Müllensiefen et al. [10] asked 1,787 participants to complete online questionnaires that measured their daily INMI experiences and OC tendencies, as well as their musical behaviors and expertise. OC tendencies were measured with a standardized obsessive compulsion inventory (OCI-R) [22]. An analysis using structural equation modeling showed that high OC tendencies were positively related to the frequency and disturbance of INMI; however, they were not directly related to the unpleasantness of INMI. Floridou et al. [11] also examined the relationship between OCI-R scores and the characteristics of INMI experiences using an online survey. They found that while the OCI-R scores correlate only partially with the frequency of INMI experiences, the scores of all subscales in OCI-R (washing, obsessing, hoarding, checking, and neutralizing) were positively related to the negative valence of INMI experiences. Although there are small discrepancies, these studies have shown that individuals with high OC tendencies experience INMI more frequently and perceive the experiences as less positive.

However, one general concern about these studies is that they examined the characteristics of INMI using retrospective self-reporting measures. INMI often occurs without realization and can be said to be on the fringe of consciousness. Therefore, retrospective self-reporting does not necessarily capture the actual characteristics of INMI in daily life. Instead, the responses are more likely to reflect characteristics of previous INMI experiences that tend to be remembered. For example, disturbing INMI experiences are more prone to be recalled, and their features were likely to be reflected in the self-reports. Therefore, for an accurate examination of the relationship between individual traits and the characteristics of INMI experiences, it is important to measure INMI using methods that do not rely solely on memory recall but instead assess these experiences in real time as they occur in daily life.

In the present study, we used the experience sampling method (ESM) [23] for measuring the characteristics of INMI experiences in daily life. Participants received a cue signal on their smartphones at random times during the day and were instructed to report whether they had experienced INMI, and if experienced, to describe the emotional characteristics of the INMI event. Compared to retrospective self-reporting, ESM is less affected by recollection bias and thus more suitable for investigating less noticeable experiences such as INMI. In fact, Cotter and Silvia [24] directly compared the reports from a retrospective INMI measure and a week of ESM data, both of which were obtained from the same participants, and found that the retrospective reports for the qualities of INMI experiences were unrelated to those in the ESM reports. This finding suggests that there is a gap between how people actually experience INMI in daily life and their beliefs or recollections about their experiences. In sum, INMI is better captured by ESM.

ESM has already been used in previous INMI studies. For example, Beaty et al. [25], and Cotter and Silvia [24] examined how the musical expertise of participants is related to the characteristics of INMI as measured through a seven-day ESM. Both studies showed that participants who were music majors experienced more INMI events during the sampling period than non-majors. Beaty et al. [25] also examined the relationship between the Big Five personality traits and ESM data on INMI. They found that both neuroticism and openness-to-experience are associated with the frequency of INMI, and openness-to-experience is also associated with positive evaluations for INMI. Furthermore, ESM has another advantage in that it is able to obtain contextual data, such as mood and activities in which participants were engaged at the time when they experience INMI. ESM studies using this advantage have found that INMI occurs more frequently when people feel happy [25, 26].

As previously described, studies using ESM have found that both musical expertise and personality traits will influence INMI, and these results were generally congruent with those obtained in studies that relied on self-reporting measures [2, 10, 11, 27]. However, no ESM studies have examined the relationship between OC tendencies and INMI experiences. In this study, therefore, we investigated the influence of OC tendencies on the frequency and emotional characteristics of INMI using a seven-day ESM survey. The effects of personality traits, musical expertise, and the context of INMI occurrences were also examined to confirm the findings of previous studies [24–26].

## Method

### Participants

We selected 101 undergraduate and graduate students (58 females, 43 males) who regularly use an iPhone or Android smartphone to participate in the study. All were native Japanese speakers, and their ages ranged from 18 to 24 years old (*M* = 20.98, *SD* = 1.46). The sample size was determined by referring to a previous study by Beaty et al. [25] that examined the relationship between personality traits and INMI experiences using ESM for 104 participants. Written informed consent was obtained from all participants, and they received a \1,000 bookstore gift card on the first day as a token of appreciation for their participation. This study was reviewed and approved by the ethics committee of the Department of Educational Psychology, Tokyo Gakugei University.

### Procedure

On the first day, participants completed three questionnaires regarding their individual traits (OC tendencies, personality traits, and musical expertise), after which the ESM survey was explained. Sessions were divided into groups of three to nine students. The definition of INMI was explained to the participants, as the term “earworm”–instead of INMI–defined as follows: “a phenomenon in which an excerpt of music comes to mind spontaneously and is then continuously repeated in one’s head irrespective of intention.” Participants were then asked to rate their understanding of this definition using a five-point rating scale of 1 (“not at all”) to 5 (“very well”). None of the participants reported options 1 (“not at all”) or 2 (“not well”). They were then asked to complete the three questionnaires, and finally, they installed the app used for the ESM survey onto their smartphones and were instructed about its usage and response procedures.

On the second day, participants began a seven-day ESM survey. Each day they received randomly timed cue signals on their smartphone as they went about their regular lives. Whenever they realized a cue had arrived, they answered questions about the occurrence of INMI and its emotional valence as well as questions about their current mood and activity. The following are the details of each questionnaire and the ESM survey.

### Questionnaire of individual traits

#### Obsessive-compulsive tendencies

The obsessive-compulsive tendency scale for Japan [28] was used to measure participants’ OC tendencies. It was based on the Maudsley Obsessional Compulsive Inventory [29], the Padua Inventory [30], and the indecisiveness scale [31]. This scale is comprised of four subscales: intrusive thoughts (6 items; e.g., “an unpleasant idea came to my head unintentionally and I was unable to stop it”), compulsive checking (6 items; e.g., “I carefully check a letter many times before posting it”), indecisiveness (6 items; e.g., “it always takes time to make decisions”), and compulsive washing (6 items; e.g., “I feel dirty when I touch money”). Twenty-four items were printed on two sheets of A4 paper, with each item rated on a scale from 1 (“disagree”) to 5 (“agree”).

#### Personality traits

The short form of the Japanese Big Five scale [32] was used for the assessment of five personality factors. This scale was developed by selecting 29 of 60 items from the Big Five scale of personality trait adjectives [33], a scale that is commonly used for personality assessment in Japan. This scale consisted of five subscales: extraversion (5 items; e.g., “gregarious”), conscientiousness (7 items; e.g., “deliberate”), neuroticism (5 items; e.g., “anxious mind”), openness-to-experience (6 items; e.g., “progressive”), and agreeableness (6 items; e.g., “tender minded”). Twenty-nine adjectives related to personality were printed on two sheets of A4 paper. Participants were required to answer how each adjective reflected on themselves using a rating scale from 1 (“not true at all”) to 7 (“very true”).

#### Musical expertise

As in previous studies [e.g., 24], the Goldsmith Musical Sophistication Index (Gold-MSI) [34] was used. This scale was originally developed to measure a wide variety of musical skills and behaviors ranging from instrument performance and listening expertise to the ability to employ music in functional settings or to communicate fluidly about music. The 39 items of the original scale were translated to Japanese after receiving permission of the authors. The Gold-MSI consists of five subscales: active engagement (9 items), perceptual abilities (9 items), musical training (7 items), singing abilities (7 items), and emotions (6 items). This scale also measures a general sophistication score (18 items) by incorporating items from each of the five subscales. Items were rated on a seven-point scale from 1 (“completely disagree”) to 7 (“completely agree”). Alternatively, for some questions participants selected answers from among seven options, each of which represented a period or frequency of a particular musical experience or activity. The 39 sets of question and responses were printed on three sheets of A4 paper.

### ESM application and questions

ESM data collection was conducted using the Personal Analytics Companion (PACO) app (www.pacoapp.com) that was installed on every participant’s iPhone or Android smartphone. The ESM data were collected over seven days. PACO sent cue signals to participants six times per day at random intervals between 8 a.m. and 10 p.m.

In response to the arrival of a cue signal, participants activated the PACO screen and answered questions directly through the app. The first question was the following: “Can you answer questions about your state of mind and the situation you were in when the cue arrived?” If participants answered “Yes,” the second question was presented. If participants chose one of the negative options (“No, I cannot remember anything although I realized the cue arrived,” or “No, I did not realize the cue arrived”), the inquiry was stopped at that point. The second question asked participants about the occurrence of INMI: “Were you experiencing earworms the moment the cue arrived?” If the answer was “Yes,” two additional questions with seven-point scales were introduced: the first was a question regarding the pleasantness of the INMI experience, and the answers ranged from 1 (“very unpleasant”) to 7 (“very pleasant”). The second question related to the likability of the music heard internally, scored from 1 (“strongly dislike”) to 7 (“strongly like”). If the answer to the second question was “No,” the following questions were omitted. Subsequently, six contextual questions followed the INMI questions. Three questions related to the participant’s mood at the moment the cue arrived, for example, “Did you feel happy/sad/worried when the cue arrived?” The other three questions related to the activities in which the participants were engaged when the cue signal arrived. Examples included the following: “Did the activities in which you were involved require concentration?”; “Did they require effort?”; and “Did you find them difficult?” Participants answered these questions using seven-point scales scored from 1 (“not at all”) to 7 (“very much”). After completion, participants touched the “Submit” button on the PACO screen to send their answers to our data server.

Participants were asked to carry their smartphones whenever possible during the sampling period and to set the volume and vibration settings high so they could easily detect the arrival of a cue. All were previously informed that they were free to cancel their participation at any time without prior contact with us.

### Statistical analysis

The data were analyzed using multilevel models using the lmerTest package in R ver. 3.4.2 because the responses to the ESM questions (Level 1) were nested within participants (Level 2). Scores of each individual trait (OC tendencies, the Big Five personality traits, and musical expertise) were computed by averaging the responses of the subscale items and were used as Level 2 predictors in the analysis. Level 1 predictors (responses to the contextual questions) were centered at each participant’s mean, and Level 2 predictors (individual trait scores) were centered at the grand mean of all samples. The dependent variables were the occurrence of INMI, the pleasantness of the INMI experience, and the extent of likability of the music heard internally. Reporting in advance, intraclass correlation coefficients of these variables were .12 with 95% CI [.09, .17], .22 [.14, .31], and .16 [.09, .25], respectively. Because the occurrence of INMI was binary data, a multilevel logistic regression analysis was conducted using a logit link function to regress the data (*N* = 1,905, as will be noted later). The other two variables (*N* = 538) were analyzed by hierarchical linear modeling. The models were estimated using maximum-likelihood estimation. When the effect of individual traits (Level 2) was analyzed, a model with only random intercepts was examined. When the effect of Level 1 predictors was analyzed (models with single predictors were examined, as noted below), a model with both a random slope and random intercept was examined.

A power calculation for the hierarchical linear analysis was performed using the MLPowSim software package [35]. The statistical power in 101 samples was estimated for the random intercept model with five Level 2 predictors; this estimate was used for the main analysis in the present study. The standardized partial regression coefficient for the analysis was determined to be .20, based on references to previous studies [24]. The Level 1 sample size, random effect variance, and significance level were set at 20, 0.30, and .05, respectively. The calculated power was .93 for each predictor. From the power analysis for the multilevel logistic regression model with the same parameters, the power of .76 was revealed.

## Results

### Descriptive statistics

Data from nine participants who sent ESM data less than ten times over the seven days and one participant with too much missing data in the Gold-MSI were excluded from the analysis. For the remaining 91 participants, ESM inquiries were answered an average of 20.9 times (*SD* = 7.1). Accordingly, a total of 1,905 data responses were collected. INMI was reported to have been experienced in 538 cases (28.2%). The mean rate of INMI occurrence averaged across participants was .29 (*SD* = .18). Previous ESM studies reported incidences of INMI between 0.17 and 0.47 [19, 24, 25, 36]. Our value was near the mid-point of that range.

The scores of each subscale of trait measures were calculated for each participant by quantifying and averaging the responses to the questions. Score reliability (Cronbach's coefficient alpha) was good for all subscale as shown in Table 1. Table 1 also shows the mean and SD of each subscale scores across 91 participants.

**Table 1:** Mean and SD of each subscale of trait measures.

	Alpha	Mean	*SD*	Min	Max
OC tendencies (1–5)					
Intrusive thoughts	.81	2.6	0.9	1.0	4.8
Compulsive checking	.87	2.8	1.0	1.0	4.8
Indecisiveness	.64	3.2	0.7	1.7	4.7
Compulsive washing	.81	2.4	0.9	1.0	5.0
Personality traits (1–7)					
Extraversion	.89	4.6	1.4	1.0	7.0
Conscientiousness	.84	3.6	1.1	1.0	6.0
Neuroticism	.85	4.7	1.3	1.4	7.0
Openness	.80	4.0	1.0	2.0	6.7
Agreeableness	.80	4.5	1.0	1.8	7.0
Music expertise (1–7)					
General sophistication	.90	3.7	1.0	1.2	5.9
Active engagement	.85	3.4	1.2	1.0	6.0
Perceptual abilities	.88	4.5	1.0	1.9	6.3
Musical training	.78	3.0	1.2	1.0	6.1
Emotions	.74	4.4	1.0	1.7	6.2
Singing abilities	.82	4.0	1.1	1.3	6.4

### Emotional characteristics of INMI and context when INMI occurred

The responses for all INMI experiences and the contextual questions were converted to scores (1–7). Table 2 shows the mean score of each question, averaged across 538 data responses when participants experienced INMI, and 1,367 when they did not. Both the pleasantness of INMI and the likability of the music showed mean scores at or above 5.0 (i.e., 5 = “slightly pleasant/like”). More than 95% of the responses for both pleasantness and likability questions ranged from 4–7 (95.7% and 98.1%, respectively). Thus, it is safe to conclude that participants rarely felt negatively about their INMI experiences, and most were either positive or neutral to varying degrees.

**Table 2:** Mean scores of responses for questions about INMI answered during the seven-day sampling period.

		Emotional characteristics of INMI		Mood when the cue arrived			Activity when the cue arrived		
	*N*	Pleasantness	Liked the music	Happy	Sad	Worried	Concentration	Effort	Difficulty
INMI	538	5.1 (1.1)	5.7 (1.1)	4.2 (1.5)	2.2 (1.5)	3.0 (1.9)	2.7 (1.8)	2.3 (1.7)	2.2 (1.6)
No INMI	1,367	–	–	3.7 (1.7)	2.2 (1.6)	2.9 (2.0)	3.2 (2.2)	2.7 (2.0)	2.5 (1.9)

Values ranged from 1 to 7. The numbers in parentheses show standard deviation.

With regard to participants’ moods when INMI occurred, the mean score of the question related to happiness was slightly higher than the score when participants had not experienced INMI. The scores for “sad” and “worried” did not fluctuate based on the presence or absence of INMI. As for the type of activities when INMI occurred, the mean scores were lower for all “concentration,” “effort,” and “difficulty” questions compared to responses when INMI did not occur.

### The effects of obsessive-compulsive tendencies, personality traits, and musical expertise on INMI

The effects of OC tendencies on each INMI occurrence, pleasantness of INMI, and the extent the music was liked, were all analyzed by using the scores of four OC subscales (intrusive thoughts, compulsive checking, indecisiveness, and compulsive washing) as predictors. As shown in Table 3, OC tendencies were shown to influence both the occurrence and emotional characteristics of INMI. Significant effects of intrusive thoughts were observed on INMI occurrence (*B* = 0.47, *p* < .001). Compulsive washing had a negative effect on the pleasantness of INMI experiences (*B* = -0.25, *p* = .001), that is, participants with a higher washing tendency felt that INMI events were less pleasant. As for the extent that the internal music was liked, significant negative effects were observed in both compulsive checking (*B* = -0.16, *p* = .021) and compulsive washing (*B* = -0.19, *p* = .005).

**Table 3:** Effects of obsessive-compulsive tendencies, personality trait, and musical expertise.

	INMI occurrence				Pleasantness				Liked the music			
	βx	*B*	*SE*	Wald *z*	β	*B*	*SE*	*t*	β	*B*	*SE*	*t*
OC tendencies												
Intrusive thoughts	**0.42**	**0.47**	**0.13**	3.59***	-.01	-0.01	0.09	-0.08	-.01	-0.01	0.08	-0.13
Compulsive checking	-0.12	-0.12	0.12	-1.06	-.01	-0.02	0.08	-0.22	**-.14**	**-0.16**	**0.07**	-2.35*
Indecisiveness	-0.07	-0.10	0.15	-0.66	-.05	-0.07	0.10	-0.71	.05	0.07	0.09	0.84
Compulsive washing	0.05	0.05	0.12	0.47	**-.22**	**-0.25**	**0.07**	-3.32**	**-.17**	**-0.19**	**0.07**	-2.91**
Personality traits												
Extraversion	0.13	0.09	0.09	0.98	.09	0.07	0.06	1.19	.11	0.08	0.05	1.64
Conscientiousness	-0.03	-0.03	0.11	-0.26	.06	0.06	0.07	0.87	-.09	-0.09	0.06	-1.55
Neuroticism	**0.24**	**0.18**	**0.09**	1.96*	.02	0.02	0.06	0.34	.10	0.08	0.05	1.63
Openness	0.10	0.10	0.13	0.83	.08	0.09	0.08	1.18	**.13**	**0.14**	**0.07**	2.10*
Agreeableness	0.02	0.02	0.13	0.16	.01	0.01	0.08	0.17	.06	0.07	0.07	1.00
Music expertise												
General sophistication	**0.55**	**0.52**	**0.10**	5.23***	.03	0.03	0.08	0.43	.05	0.05	0.07	0.77
Active engagement	0.07	0.06	0.12	0.48	-.06	-0.06	0.09	-0.67	.05	0.05	0.08	0.66
Perceptual abilities	0.00	0.00	0.14	-0.02	.05	0.05	0.10	0.53	-.05	-0.06	0.09	-0.65
Musical training	0.06	0.05	0.09	0.51	.01	0.01	0.07	0.09	-.02	-0.01	0.06	-0.23
Emotions	0.18	0.19	0.15	1.21	.07	0.09	0.12	0.74	.14	0.18	0.10	1.70
Singing abilities	**0.38**	**0.32**	**0.13**	2.43*	-.01	-0.01	0.10	-0.12	-.04	-0.04	0.09	-0.44

β = standardized fixed effect estimate (βx was estimated by only the predictors were standardized). *B* = fixed effect estimate, *SE* = standard error of unstandardized fixed effect.

*** *p* < .001

** *p* < .01

* *p* < .05

The effects of personality traits on each of the three dependent variables were analyzed using Big Five subscale scores as predictors. Neuroticism significantly predicted the occurrence of INMI (*B* = 0.18, *p* = .049), that is, participants with higher neuroticism experienced more instances of INMI during the sampling period. As for the emotional characteristics of INMI, no personality trait effects were observed in the pleasantness of INMI, while the effects of openness-to-experience were significant on the extent to which the music was liked (*B* = 0.14, *p* = .039).

The degree of musical expertise was assessed using Gold-MSI. As this included the general sophistication score and five subscales, the analysis using the general sophistication score as a predictor and another using subscale scores were conducted separately. As shown in Table 3, the multilevel analysis showed a significant effect of general sophistication on the INMI occurrence (*B* = 0.52, *p* < .001). Among the five subscales, only singing ability significantly predicted the occurrence of INMI (*B* = 0.32, *p* = .015). Conversely, there were no significant effects of musical expertise on either pleasantness of INMI or the likability of the music.

In order to verify the robustness of the aforementioned effects, the analyses using all of the factors as predictors were conducted, with the exception of general musical sophistication. Table 4 shows the results of these analyses. For the analysis of occurrence of INMI, both the effects of intrusive thoughts on OC tendencies (*B* = 0.40, *p* = .004) and singing ability (*B* = 0.38, *p* = .003) remained significant, whereas the effect of neuroticism in the Big Five disappeared (*B* = 0.12, *p* = .247). In the analysis for the pleasantness of INMI experiences, the effect of compulsive washing remained significant (*B* = -0.24, *p* = .004). Finally, in the analysis of the extent to which the music was liked, although the effect of compulsive checking did not reach significance (*B* = -0.11, *p* = .100), both the effects of compulsive washing (*B* = -0.16, *p* = .028) and openness-to-experience (*B* = 0.15, *p* = .047) remained significant.

**Table 4:** Effects of obsessive-compulsive tendencies, personality trait, and musical expertise, which were analyzed simultaneously.

	INMI occurrence				Pleasantness				Liked the music			
	βx	*B*	*SE*	Wald *z*	β	*B*	*SE*	*t*	β	*B*	*SE*	*t*
OC tendencies												
Intrusive thoughts	**0.35**	**0.40**	**0.14**	2.90**	.04	0.05	0.11	0.45	-.01	-0.02	0.10	-0.19
Compulsive checking	-0.16	-0.17	0.10	-1.62	-.03	-0.04	0.08	-0.50	-.10	-0.11	0.07	-1.66
Indecisiveness	-0.07	-0.10	0.16	-0.63	.04	0.05	0.12	0.46	.08	0.11	0.10	1.07
Compulsive washing	0.05	0.05	0.10	0.51	**-.21**	**-0.24**	**0.08**	-3.01**	**-.14**	**-0.16**	**0.07**	-2.24*
Personality traits												
Extraversion	-0.12	-0.09	0.08	-1.09	-.01	0.00	0.06	-0.08	.05	0.04	0.05	0.65
Conscientiousness	-0.01	-0.01	0.10	-0.14	.09	0.09	0.07	1.26	-.03	-0.03	0.06	-0.40
Neuroticism	0.16	0.12	0.10	1.16	-.06	-0.05	0.08	-0.66	.03	0.03	0.07	0.36
Openness	-0.19	-0.19	0.11	-1.70	.12	0.13	0.09	1.54	**.14**	**0.15**	**0.08**	2.02*
Agreeableness	0.14	0.15	0.12	1.27	-.02	-0.03	0.09	-0.27	.02	0.03	0.08	0.35
Music expertise												
Active engagement	0.00	0.00	0.12	0.00	-.04	-0.04	0.09	-0.41	.01	0.01	0.07	0.15
Perceptual abilities	0.05	0.05	0.12	0.39	.05	0.06	0.09	0.62	-.07	-0.07	0.08	-0.92
Musical training	0.21	0.17	0.09	1.89	-.01	-0.01	0.07	-0.15	.00	0.00	0.06	-0.05
Emotions	0.12	0.13	0.14	0.92	.03	0.03	0.11	0.30	.08	0.11	0.10	1.11
Singing abilities	**0.45**	**0.38**	**0.13**	3.01**	-.04	-0.05	0.10	-0.50	-.04	-0.04	0.08	-0.49

β = standardized fixed effect estimate (βx was estimated by only the predictors were standardized). *B* = fixed effect estimate, *SE* = standard error of unstandardized fixed effect.

*** *p* < .001

** *p* < .01

* *p* < .05

### The effects of moods and activities on INMI

Since the score for each mood (i.e., happy, sad, and worried) was expected to correlate with each other, their effects were analyzed separately. As shown in Table 5, though neither the effect of a happy, sad, nor worried mood was significant for the occurrence of INMI, the effects of all three moods were significant for the pleasantness of INMI (*B* = 0.29, *B* = -0.20, and *B* = -0.11, respectively; *ps* < .001). The effect of happy moods was also observed for likability of the music (*B* = 0.13, *p* < .001).

**Table 5:** Effect of mood and activity on INMI.

	INMI occurrence			Pleasantness			Liked the music		
	*B*	*SE*	Wald *z*	*B*	*SE*	*t*	*B*	*SE*	*t*
Happy	0.07	0.05	1.54	**0.29**	**0.04**	7.73***	**0.13**	**0.03**	3.91***
Sad	-0.05	0.06	-0.90	**-0.20**	**0.04**	-5.24***	-0.02	0.04	-0.59
Worried	-0.05	0.05	-1.02	**-0.11**	**0.03**	-3.50***	-0.02	0.03	-0.65
Concentration	**-0.14**	**0.04**	-3.59***	-0.03	0.03	-1.08	-0.01	0.03	-0.22
Effort	**-0.10**	**0.04**	-2.50*	-0.02	0.04	-0.53	0.02	0.03	0.68
Difficulty	**-0.11**	**0.04**	-2.58*	-0.03	0.03	-0.96	0.02	0.03	0.74

*B* = fixed effect estimate, *SE* = standard error of fixed effect.

*** *p* < .001

** *p* < .01

* *p* < .05

For the scores of each activity question (i.e., concentration, effort, and difficulty), as in the case of the mood scores, the scores were analyzed separately. All three activity scores showed significant effects on the occurrence of INMI (*B* = -0.14, *p* < .001 for concentration, *B* = -0.10, *p* = .012 for effort, and *B* = -0.11, *p* = .01 for difficulty), but no effect was observed in the analysis of the pleasantness of INMI or the extent to which the music was liked.

In addition, to examine whether the effect of intrusive thoughts on the occurrence of INMI differed according to mood or activities when the cue arrived, the interactions between intrusive thoughts and each of the above six Level 1 variables were analyzed. However, no significant interaction was observed in any of these examinations. Furthermore, the interactions between compulsive washing and these six variables in the analyses regarding the pleasantness of INMI and the extent to which the music was liked were examined. Again, no significant interaction was observed, except for the interaction between compulsive washing and sad moods for the pleasantness of INMI (*B* = 0.09, *SE* = 0.04, *t* = 2.13, *p* = .035), indicating that the effect of sad moods was greater for participants with low washing tendencies.

### The time-series variation of INMI occurrence

Finally, to examine how the occurrence frequency of INMI changed during the sampling period, we analyzed the effect of elapsed time on the occurrence of INMI. More specifically, the effects of day (1‒7) and trial (1‒42) on ESM inquiry responses were examined. A multilevel logistic regression analysis with the day and trial as Level 1 predictors was conducted separately. However, the results showed no significant effect on day (*B* = 0.01, *SE* = 0.03, Wald *z* = 0.37, *p* = .711) nor trial (*B* = -0.003, *SE* = 0.01, Wald *z* = -0.42, *p* = .672).

## Discussion

The present study examined the effect of OC tendencies on emotional characteristics and the frequency of involuntary musical imagery (INMI). Previous studies using retrospective self-reporting have suggested a relationship between OC tendencies and INMI [10, 11]. We examined the validity of these findings using an experience sampling method (ESM) that measures the characteristics of INMI experiences in real time at the moment they occur in daily life. The effects of the Big Five personality traits and musical expertise on INMI were also examined. INMI was reported to occur with a probability of about 30% during the seven-day sampling period. OC tendencies positively affected occurrences (i.e., frequency) of INMI and negatively affected both the pleasantness of INMI experiences and the extent of likability of the music heard internally. The effects of personality traits and musical expertise were also significant in the analysis of INMI occurrences. In addition, the effects of moods and activities at the time of INMI occurrence were observed.

Before considering the implications of these results, we should discuss the validity of the responses obtained from ESM. ESM has a possible weakness in that the repeated assessments lead participants to pay unusual attention to their internal states [37]. In light of this, repeated sending of ESM inquiries might have increased participants’ attention to music and made them more likely to experience INMI. Therefore, there is the possibility that the characteristics of INMI measured in this study were specific to ESM and might differ from those experienced in daily lives. We verified this possibility by analyzing time-series variation in INMI occurrence. If this possibility were the case, one would expect more INMI to be reported later in the sampling period than earlier. However, the analysis showed no effect of day nor trial on INMI occurrence. This result suggests that the reports of INMI experiences were unlikely to be affected by repeated answering about INMI. Therefore, we can safely discuss the implications of each result shown by ESM.

### Effects of obsessive-compulsive tendencies

Prior studies [10, 11] have reported that persons who are high in OC tendencies experienced more INMI and considered them as less positive experiences than those with low OC tendencies. However, as these findings were obtained by using retrospective self-reports that are shown to be susceptible to recollection bias, they required further validation through the use of more appropriate methods. The present study used ESM and mostly supported the findings in the two aforementioned studies.

Regarding the effects of OC tendencies on INMI occurrences, significant positive effects were observed for intrusive thoughts, whereas the other subscale scores had no effects. In the study of Floridou et al. [11], only obsessing (intrusive thoughts) and neutralizing showed significant correlations with the frequency of INMI among the subscales of OCI-R. In the study of Müllensiefen et al. [10], obsessing also showed the highest correlation with INMI frequency. Therefore, the results of our study are completely congruent with the findings in these previous studies. The term “intrusive thoughts” refers to unpleasant thoughts that repeatedly enter one’s mind irrespective of intention. Meanwhile, it is shown that people who frequently experience INMI were also prone to experiencing a broad range of involuntary thoughts, including memories of the past as well as thoughts about the future, friends, and work [38]. Therefore, this general tendency to think involuntarily, possibly caused by failures of executive control [39], might mediate the relationship between the high frequency of INMI and the tendency toward intrusive thoughts.

One objection for this interpretation would be that the intrusive-thoughts subscale partially measured the frequency of INMI in daily lives as repetitive “thought”, along with that of the other negative thoughts. If this was the case, the results explained here were not surprising because both the intrusive-thoughts subscale and the ESM questions measured the same experiences in different ways. To examine this possibility, we conducted an additional online survey on 100 participants. In this survey, the participants answered the questions of the intrusive-thoughts subscale and reported what they assumed to be the content of intrusive thoughts to answer the previous questions by selecting multiple options from eight alternatives (“past event”, “current concern”, “something or someone that one dislikes”, “music”, “visual image”, “desires and feelings”, “what someone has to do”, and “other”). The results showed that only two participants chose “music” as the content of intrusive thoughts. Therefore, the positive effect of intrusive thoughts on the occurrence of INMI cannot be explained by the possibility that the intrusive-thoughts subscales also measured the frequency of INMI.

OC tendencies were shown to have a negative impact on the emotional characteristics of INMI experiences. Specifically, compulsive washing negatively affected both the pleasantness of INMI and liking the music heard internally. Studies employing retrospective self-reports showed that OC tendencies influence the negative valence [11] and extent of disturbance of INMI [10]. Therefore, once again our study supports these findings while using a more ecologically valid method. It should be noted that these results do not imply that participants with high washing tendencies were more likely to experience unpleasant INMI. In the present study, consistent with the previous studies [7–10], almost all INMI occurrences were reported as either neutral or pleasant experiences. Therefore, to be exact, participants with high washing tendencies experienced INMI as “less pleasant” than those with low washing tendencies.

There was one discrepancy between the present and previous studies. In the present study, only compulsive washing affected the pleasantness and music likability scores in INMI. In the survey of Floridou et al. [11], all subscales of OCI-R were correlated with the negative valence of INMI. Considering that our method can be regarded as more appropriate for measuring INMI, the current conclusion is that the emotional characteristics of INMI are at least related to the tendencies of compulsive washing; however, their relationship to other OC attributes remains unclear. Persons with high washing tendencies experience feelings of anxiety and disgust that are repeatedly evoked by intrusive thoughts about contamination [40]. Based on this, we speculate that persons with high washing tendencies may feel uncomfortable with the repetition of ideas or images occurring in their minds without their conscious control even if they are not related to dirt. This tendency may reduce the pleasantness of INMI even though the original experience may be considered pleasant. In addition, a recent neuroscientific study showed that persons who experienced INMI as less positive, have larger cortical volume in the para-hippocampal region [12]. This region is known to be involved in affective evaluation of stimuli [41] and is also found to show greater activity in OCD patients with high washing tendencies [42]. Therefore, there is a possibility that the relationship between high washing tendencies and less positive feeling with INMI stems from this cortical characteristic of the para-hippocampal region.

### Effect of personality traits

A positive effect of neuroticism on the occurrence of INMI was observed, that is, participants high in neuroticism were more likely to experience INMI. This finding is consistent with the earlier study of Beaty et al. [25] in which both the retrospective survey and ESM data showed a positive effect of both neuroticism and openness-to-experience. However, in the present study, when the regression analysis was conducted with the OC tendencies, the effect of neuroticism disappeared while the effect of intrusive thoughts remained intact. Previous studies have reported that patients diagnosed with OCD have higher degrees of neuroticism [43], and intrusive thoughts (obsession) are more strongly related to neuroticism than the other symptoms of OCD [44]. In fact, the correlation analysis of the present data showed moderate correlations between neuroticism and scores for each subscale of OC tendencies (*r* = .36 –.55, *ps* < .005) except for compulsive washing (*r* = -.004, *p* = .972) (see the table in S1 File). This result suggests that the relationship between INMI occurrence and neuroticism observed in this and previous studies reflected the relationship between INMI occurrence and susceptibility to intrusive thoughts, with the latter a known characteristic of persons with high neuroticism.

Regarding the relationship between personality traits and the emotional characteristics of INMI, no personality trait affected the pleasantness of INMI experiences, whereas only openness-to-experience showed a positive influence on liking the music. In prior studies, while Cotter et al. [27] reported no effect of personality traits on the negative valence of INMI, Beaty et al. [25] found that openness-to-experience positively affected the likability of INMI experiences. In the latter study, participants were asked to express the extent of their likability. Given these similarities, openness-to-experience may affect the likability for experiences related to INMI rather than its pleasantness or emotional valence.

### Effect of musical expertise

As with the previous studies [10, 24], musical expertise did not affect the emotional characteristics of INMI. On the other hand, the general musical sophistication score of Gold-MSI positively affected the occurrence of INMI. This finding aligns with previous studies that were based on retrospective self-reports [2, 10, 11] and ESM [24] that found that persons with high musical expertise were more likely to experience INMI. Furthermore, among the subscales of Gold-MSI, only singing ability was shown to have a positive influence on INMI occurrences. This result is consistent with the findings from an online survey by Müllensiefen et al. [10] who reported that singing ability positively affected the length of INMI despite no direct relationship to INMI frequency being observed in the study. In an experimental study, Beaman et al. [45] reported that interference with articulatory motor programming by chewing gum reduced the occurrences of INMI. This finding suggests that INMI is based on vocal and subvocal systems that are also used for singing and humming [46]. Therefore, it is not surprising that there is a relationship between singing ability and INMI. However, the other potential explanation for this result may be that participants with advanced singing ability were more likely to intentionally sing songs internally to themselves as practice or enjoyment [47]. In this study, voluntary and involuntary music imagery were not strictly separated. The relationship between singing ability and INMI frequency should be further investigated by distinguishing the intentionality of music imageries.

### Effects of mood and activity

The analysis for the effect of mood on INMI occurrences did not show a significant positive effect for happy moods, although the mean score for the question about happiness was slightly higher when participants had experienced INMI. Similarly, neither the effects of sadness nor worry were significant in this study. These results are inconsistent with findings of previous ESM studies [25, 26]. Therefore, the effect of mood on the occurrence of INMI requires further verification.

With respect to the pleasantness of INMI experiences, all the effects of happiness, sadness, and worrisome moods were significant. In general, positive moods were correlated with the pleasantness of INMI experiences. There are two possible explanations for this finding: positive moods made participants experience INMI as more pleasant, and INMI might have an emotion regulation function [36], that is, the occurrence of pleasant INMI may make participants feel positive. This is also true for the significant positive effect of happy moods on the extent to which the participants liked the music. This may indicate a mood congruency effect where participants were prone to hear likable songs when they felt happier, or it may suggest that hearing favorite songs internally would naturally increase happiness. In relation to this question, Floridou and Müllensiefen [19] analyzed ESM data using Bayesian network modeling and found no direct effect of INMI on mood, thereby suggesting that the former view is more plausible. Nonetheless, the causal relationship between mood and emotional characteristics of INMI should be further investigated using experimental methods.

As for the effect of activities in which participants were engaged during INMI, the results showed that INMI experiences were more likely to occur when participants were engaged in activities that require less concentration and effort and are less difficult. INMI is more likely to occur during activities performed with a low cognitive load; this has previously been found in studies using a diary method [9] and those for laboratory-induced INMI [9, 18]. These findings suggest that INMI occurs when executive control works weakly, as explained in the Introduction, and is consistent with the view that both INMI and intrusive thoughts of OCD [20, 21] occur as the result of faulty executive control.

## Conclusion

The present study investigated the influence of OC tendencies, personality traits, and music expertise on the frequency and emotional characteristics of INMI using ESM, a method proven to be appropriate for recording the characteristics of INMI occurrences in real time. INMI has been shown to provide generally pleasant experiences; however, the degree of pleasantness varies among individuals. The importance of this study is to clarify that OC tendencies, in particular the compulsive washing tendencies, affect these individual differences. Another important finding is the confirmation that the tendencies of intrusive thoughts affect INMI occurrence. Moreover, the results of the effects of personality traits, music expertise, and contextual factors mostly supported the previous studies. In future research, the underlying mechanisms of these relationships should be further clarified.

## Supporting information

S1 Dataset ESM (INMI occurrence) and questionnaire data. (XLSX)

S2 Dataset ESM (pleasantness and liked the music) and questionnaire data. (XLSX)

S1 File Coefficients between trait scores. (XLSX)
